# The Support to Rural India's Public Education System (STRIPES) Trial: A Cluster Randomised Controlled Trial of Supplementary Teaching, Learning Material and Material Support

**DOI:** 10.1371/journal.pone.0065775

**Published:** 2013-07-16

**Authors:** Rashmi Lakshminarayana, Alex Eble, Preetha Bhakta, Chris Frost, Peter Boone, Diana Elbourne, Vera Mann

**Affiliations:** 1 Effective Intervention, Centre for Economic Performance, London School of Economics, London, United Kingdom; 2 Department of Economics, Brown University, Providence, Rhode Island, United States of America; 3 The Naandi Foundation, Banjara Hills, Hyderabad, India; 4 Department of Medical Statistics, The London School of Hygiene and Tropical Medicine, London, United Kingdom; 5 Department of Medical Statistics, The London School of Hygiene and Tropical Medicine, London, United Kingdom; UC Davis School of Medicine, United States of America

## Abstract

**Background:**

The aim of the STRIPES trial was to assess the effectiveness of providing supplementary, remedial teaching and learning materials (and an additional ‘kit’ of materials for girls) on a composite of language and mathematics test scores for children in classes two, three and four in public primary schools in villages in the Nagarkurnool division of Andhra Pradesh, India.

**Methods:**

STRIPES was a cluster randomised trial in which 214 villages were allocated either to the supplementary teaching intervention (n = 107) or to serve as controls (n = 107). 54 of the intervention villages were further randomly allocated to receive additional kit for girls. The study was not blinded. Analysis was conducted on the intention to treat principle, allowing for clustering.

**Results:**

Composite test scores were significantly higher in the intervention group (107 villages; 2364 children) than in the control group (106 villages; 2014 children) at the end of the trial (mean difference on a percentage scale 15.8; 95% CI 13.1 to 18.6; p<0.001; 0.75 Standard Deviation (SD) difference). Composite test scores were not significantly different in the 54 villages (614 girls) with the additional kits for girls compared to the 53 villages (636 girls) without these kits at the end of the trial (mean difference on a percentage scale 0.5; 95% CI -4.34 to 5.4; p = 0.84). The cost per 0.1 SD increase in composite test score for intervention without kits is Rs. 382.97 (£4.45, $7.13), and Rs.480.59 (£5.58, $8.94) for the intervention with kits.

**Conclusions:**

A 18 month programme of supplementary remedial teaching and learning materials had a substantial impact on language and mathematics scores of primary school students in rural Andhra Pradesh, yet providing a ‘kit’ of materials to girls in these villages did not lead to any measured additional benefit.

**Trial Registration:**

Controlled-Trials.com ISRCTN69951502

## Introduction

Effective provision of education in rural areas of the developing world is an issue which has troubled policymakers, activists, and scholars for decades [1], [2]. India has struggled with this problem since its independence and, despite recent progress, there remain hundreds of millions of Indians with little to no education. A recent survey of education levels in India documents an increase in the number of five year olds enrolled in schools from 54.9% in 2009 to 62.8% in 2010, but also reports that even after five years of schooling, more than half (53.4%) of all children surveyed still attending school at the fifth class could not read, write or solve arithmetic problems expected of children in the second class [3]. There are several explanations for these low learning levels: high levels of teacher absenteeism, low teacher effort levels when teachers are in class, and a disconnect between parents and educational providers [4], [5]. The Indian government has attempted to address these issues in education with programmes such as *Sarva Siksha Abhiyan* [6], however, there has been no rigorous evaluation of the impact of this intervention [7].

In the last decade there has been a spate of research attempting to evaluate the efficacy of interventions which increase either the quantity or the quality of public education or which stimulate demand for education through incentive programmes. A review study [8] identifies a series of interventions, such as merit scholarships, teacher monitoring programmes, school health programmes, provision of uniforms to girls, conditional cash transfers to parents, and supplementary education programmes, which have succeeded in raising both attendance and performance levels in rural schools across the developing world.

A few studies reviewed [8] have evaluated the effect of increasing the quality or quantity of education supplied on learning levels. One trial evaluated an education programme which hired and trained a young woman from the community to provide remedial support to low performing children in classes 3 and 4, and found an increase in average test scores in treatment schools relative to controls by 0.14 standard deviations (SD) in the first year and 0.28 SD in the second year [9]. Another randomised trial evaluated a teacher performance pay scheme across a large representative sample of government-run rural primary schools in Andhra Pradesh and found that after two years of the programme, students in incentive schools performed better than those in control schools by 0.27 SD and 0.17 SD in maths and language tests, respectively [10].

Within this growing body of evidence, there remain three major gaps in the literature. One, there is little evidence evaluating on-going programmes implemented by local NGOs, as opposed to novel programmes designed specifically for a given study often with only short-term piloting of the intervention before the trial begins. Two, there are few studies which attempt to replicate the efficacy results published to date, as publication bias favours new interventions and findings. Finally, there is even less evidence which evaluates educational interventions operating in particularly poor and remote areas of India. Our study was implemented as an attempt to address each of these gaps.

The Naandi Foundation (henceforth “Naandi”), a large Indian NGO, has been implementing education programmes similar to those discussed above for several years and has expanded them to several states in India. The overarching goal of the programme is to ensure that every underprivileged child gets the academic and social support necessary to complete 10 years of schooling. One prong of this work is the Ensuring Children Learn (ECL) initiative, which provides after-school instruction in government primary schools in rural and urban areas focusing on remedial maths and language skills. Another intervention of interest is the *Nanhi Kali* programme which provides material support for girls in the form of school uniforms and school bags in addition to the academic support provided in the ECL programme. The STRIPES trial was designed to evaluate the impact of these two programmes.

The STRIPES trial was embedded within the CHAMPION trial which evaluates a programme of community health education for mothers, safe home deliveries and contracting out to the private sector for complicated deliveries. The control group for the CHAMPION trial was the intervention group for the STRIPES trial (and vice versa). The aim of the STRIPES trial was to evaluate the impact of educational support on children's learning. An additional comparison assessed the value of providing additional material support for girls. As both interventions were provided at the village level, the primary units for randomisation were the villages. Given the focus of the CHAMPION trial was on pregnant women and neonates, and the focus of the STRIPES trial was on children in primary school, we believed there would be little risk of one intervention having an impact on the outcomes of the other.

## Methods and Outcomes

The reporting for the STRIPES trial follows the CONSORT guidelines for cluster randomised controlled trials [11]. The protocol for this trial and supporting CONSORT checklist are available as supporting information; see Checklist S1, Checklist S2 and Protocol S1.

### Objectives

The primary objectives of this study were to (i) assess the effectiveness of a widely used NGO intervention, providing supplementary remedial teaching and learning materials to children in classes 2–4 in public primary schools in villages in Andhra Pradesh, on their language and maths scores evaluated after two academic years of the programme (comparison 1); (ii) assess the effectiveness of the intervention in (i) alongside additional material support provided to girls, relative to the intervention without this additional support, on girls' performance in the same classes over the same time period (comparison 2).

The main secondary objectives were to assess the cost per child of the supplementary teaching and learning materials programme when implemented in this rural setting, and to assess the costs relative both to the benefits of the additional material support provided to girls in this intervention.

### Participants

The trial was conducted in villages with a population of less than 2,500 people in the Nagarkurnool division in the state of Andhra Pradesh in India which were participating in the CHAMPION Trial [12]. All children living in these villages who were potentially eligible for the trial were listed in January 2008, before the randomisation for the Champion trial. This enumeration was based on information given by any persons who were present in the households at the time. Baseline tests for maths and language were conducted between September and November of 2008. The interventions took place from December 2008 to April 2010. An endline evaluation was conducted in May of 2010.

At the start of the trial, a survey team collected background information on each school and village including the number of girl and boy students in classes two, three, and four at each school in eligible villages, the number of teachers in each school, the number of blackboards (collected as a proxy for the overall quality of school infrastructure), and whether the village was tribal or non-tribal.

A village was eligible for inclusion if it:

- was already participating in the CHAMPION Trial
- had at least one public primary school in the village serving boys and girls
- this school operated in the 2007–08 academic year and was likely to continue operations during the following two years
- at least 15 children in total were present in classes two, three, and four in the school at the time of the baseline test [13]

A child was eligible for inclusion in the analysis of the trial if s/he satisfied the following criteria:

- S/he was resident in an eligible village
- S/he was recorded in the enumeration conducted in January 2008 (described in further detail below) as planning to be enrolled in the 2^nd^, 3^rd^ or 4th class at the government school located in her/his village in the 2008–9 academic year
- After hearing an explanation of the trial, her/his parent(s) or guardian(s) did not choose to opt out of the trial.

### Ethics

The consent process initially followed that for the CHAMPION Trial [12] and is described in the trial protocol [13]. Approval of the protocol was obtained from the Department of Education of the Government of Andhra Pradesh. Consent was obtained from the Panchayat (the smallest democratically elected unit of government in rural India). Members of the trial team explained to each Panchayat the two interventions, health and education, the process of randomisation, and what participating in the trial entailed for the Panchayat. The villagers gave consent both orally and in writing through the signatures of the Panchayat leaders. This process of obtaining consent through meetings with approval of the ‘guardians’ of the clusters is common in trials in which the intervention is delivered at the level of a cluster [14], [15]. Further consent was obtained from the Panchayats to conduct the second randomisation, which randomly allocated villages in the treatment arm to receive or not receive additional material support for girls.

Members of the intervention team informed parents or guardians of children about the trial in both STRIPES arms prior to delivery of the interventions and explained that they had the opportunity to opt out of the trial. Parents had the option to opt out for both the instructional intervention and the additional materials for girls. If a parent chose not to allow her/his child to participate in the trial, her/his child's name was removed from the testing rolls. During testing, children in both trial arms were informed that all tests are voluntary and that they may opt out of tests if they choose to. The “opt out” method of parental permission is considered to be an ethical way of informing participants in low-risk interventions. To encourage participation and to reduce biased post-randomisation sample attrition, it was announced that all test takers would be given a pencil, sharpener, eraser, ruler and notebook.

The CHAMPION/STRIPES trials and consent procedures received ethical approval from the IRB at the LV Prasad Eye Institute, Hyderabad, India which is affiliated with the Indian Council of Medical Research (Reference number: LEC07002) in July 2007, with amendment in January 2010, and from the ethics committee at the London School of Hygiene and Tropical Medicine (LSHTM) (Reference number 5166) in June 2007, with amendment in December 2009.

### Interventions

#### 1. Supplementary teaching and learning material

In each eligible village, the field workers first engaged in an outreach programme to involve the recipient community in selection of the intervention teacher and to promote education as a common value. The team organized a community meeting at a village where in which parents in villages were mobilized to suggest and then select a Community Volunteer (CV). The CV was required to have completed 10^th^ class, when possible, and be resident in the village receiving the intervention. Once selected, the CV was trained by the Naandi Education Research Group team to deliver supplementary lessons focusing on remedial education to all children in classes two, three, and four in the first year of the trial, and to all children in classes three, four and five in the second year. To ensure children attend these lessons, the CV conducted an outreach programme in which families of eligible children entered oral agreements with the CV, promising that they would ensure that their children attend the supplementary education programme. This process of community involvement was intended to galvanise families to take responsibility for their children's attendance and performance in school.

For two academic years, the CV provided remedial instruction for two hours per day, in schools, after normal school hours, on a daily basis using principles of Cooperative-Reflective Learning (CRL) (for more details of CRL, see Box S1). The subject matter covered in these sessions reinforced the curriculum covered in the school and was tailored to students' class-specific needs and learning levels. Each CV was supported by a Field Coordinator (FC) who in turn was managed by a Deputy Programme Coordinator (DPC) in the field and a Programme Coordinator (PC) at the head office.

The Teaching and Learning Materials (TLM) used in the lessons had been developed and tested by education experts from both the Naandi Foundation and external consultants. A bundle of learning materials, including a pen, four pencils, two notebooks, a ruler and an eraser, was provided to each participating child for use in these supplementary classes. For more details of TLM, see Box S1.

#### 2. Additional material support for girls

For each of the 54 eligible villages in this group, the trial provided the services outlined above and, for the girl students, it also provided a kit of materials, including a pair of uniforms, shoes, socks, undergarments and a school bag, intended to improve attendance and performance in school. This intervention focused on girls because they are likely to face greater obstacles in attaining education than boys in disadvantaged rural areas such as that of our study [16].

### STRIPES Controls

In control villages, no education programme was implemented, but interventions for maternal and infant health were offered as part of the CHAMPION Trial.

### Outcomes

The primary endpoint was a composite of scores on language and maths assessments from an ‘endline’ test conducted in the spring of 2010, after the intervention had been implemented for 18 months.

There were three separate class-specific tests designed for the baseline tests and three more for the endline tests. These tests were designed by Educational Initiatives, an Indian firm that specialises in conducting educational assessments in rural and urban Indian schools. This group designed and implemented surveys for another major study on primary education conducted concurrently in Andhra Pradesh [10]. Each test used in our study had two sections, mathematics and language. Each section had three types of question: to test those competencies set out by the Andhra Pradesh State curriculum for that class, to test competencies set out by the Indian National curriculum for that class, and to test competencies that allow for comparison of test results with other evaluations conducted internationally. The baseline test included only questions evaluating competencies expected of children in the class in which they entered the trial. The endline test included questions which tested these same competencies and also had a section based on the government-specified anticipated competencies of children one class higher than at baseline. These tests were administered to all eligible children available in each village on the day of testing by an independent group, GH Consultancy Services, and GH test administrators were trained by Educational Initiatives. The Naandi intervention team were not part of the planning, design and testing process. They were also not at any of the testing sites on either the day of the baseline or endline test. Secondary endpoints included scores on language and maths assessments, separately and the average cost of the intervention per child.

Maths, language and composite scores were derived as follows:

- Maths percentage score: (points scored/maximum possible points) ×100
- Language percentage score: (points scored/maximum possible points) ×100
- Composite percentage score: (Maths percentage score + Language percentage score)/2.

### Sample size

A study evaluating a similar education intervention in urban areas found that the average test score of children receiving additional instruction rose by 0.14 SD compared to controls over a year [9]. We estimated that at least 15 children per village would take the test at the end of the trial. With an intra-cluster correlation coefficient of 0.03, 107 intervention villages and 107 control villages would give over 90% power to detect a difference of 0.14 SD in the standardised score between intervention and control villages with a conventional 2-sided significance level of 5%.

### Randomisation

Randomisation was conducted in two stages. After consent was obtained at the cluster (village) level, the first stage of randomisation allocated villages to STRIPES treatment/control (which are CHAMPION control/treatment, respectively) in February 2008. Villages were stratified according to whether their travel time to the nearest designated Non-Public Health Centre was less or greater than one hour, and also into three groups according to the “tribal” status of the village. The three tribal classifications were thanda (2–3 km from the main village with around 15 families), penta (20–30 km from the main village with around 4–5 families) and non-tribal (a main village). The 464 villages were randomised by LSHTM in a 1∶1 ratio, within each of these six strata, to receive either a health intervention (and therefore to serve as STRIPES controls) or an education intervention (and therefore to serve as CHAMPION controls). 232 villages were allocated to receive the health intervention and 232 were allocated to receive the education intervention.

**Figure 1:**
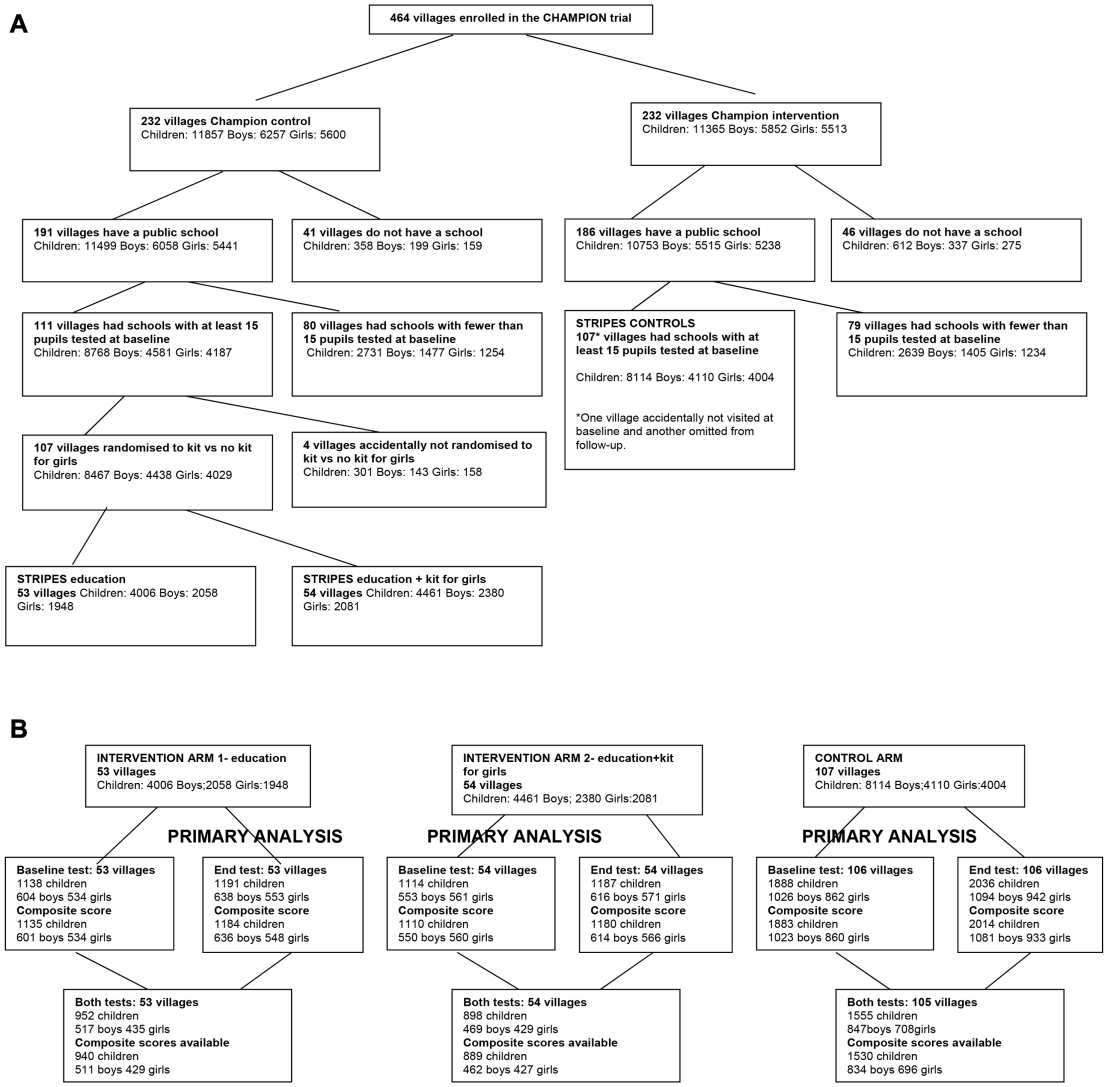
Flowchart of villages and children to the point of randomisation in the STRIPES trial, (a). Flowchart of villages and children in the analysis of the STRIPES trial, (b).

In January 2008, (prior to the first randomisation) an enumeration team used a baseline education survey to collect data about all children aged between 4 and 12 in each of the 464 CHAMPION villages. As shown in Figure 1a, of the 464 villages, 377 villages (191 CHAMPION controls; 186 CHAMPION intervention) had at least one primary public school (operating in the 2007-8 academic year and intending to operate for the duration of the trial). Of these villages, 159 (80 CHAMPION control; 79 CHAMPION intervention) had fewer than 15 children present in the village on the day of baseline testing. Children in these villages were offered the same educational support programme as trial intervention villages in the nearest intervention school, but were excluded from the trial. The remaining 218 villages were eligible for inclusion in the STRIPES trial (111 to education intervention; 107 to control). Four STRIPES intervention villages were accidentally not randomised for Comparison Two. They nevertheless received the education intervention (without the kits for girls), but were not included in the analyses. Following consent at the cluster level, the remaining 107 STRIPES intervention villages were randomly allocated in a 1∶1 ratio to either: receive supplementary teaching plus learning materials (n = 53) or supplementary teaching plus learning materials and, for girls only, additional material support (n = 54). 4006 children were in clusters allocated to receive supplementary teaching plus learning materials; 4461 to receive supplementary teaching plus learning materials plus the kit for the girls, and 8114 were STRIPES controls.

### Blinding

Owing to the nature of the interventions, this trial was an unblinded study. However, assessors were not told which the control villages were and which the intervention villages were.

### Statistical methods

The analysis was conducted according to the intention to treat principle. All enumerated children satisfying eligibility criteria were included in the primary analysis comparing (i) all STRIPES intervention children to all STRIPES control children, and (ii) all STRIPES intervention girls allocated a kit to all STRIPES intervention girls NOT allocated a kit.

Composite and individual language and maths test scores at follow-up were compared using unpaired t-tests with robust (Huber-White) standard errors allowing for clustering. Linear regression models (with robust standard errors) were used to explore the effect of adjusting for gender and baseline class as well as interactions between these factors and the intervention. As a check on robustness, we assessed the effect of the intervention using an analysis of covariance model to adjust for baseline levels in the subset of children with baseline test results.

The analyses investigating interactions between the intervention and gender were pre-specified in the protocol. The analyses investigating interactions between the intervention and baseline class were added to the statistical analysis plan after publication of the protocol.

All analyses were conducted using scores calculated on a percentage scale. We present our main estimates in terms of standard deviation scores as well as percentage scores to ease comparison with other studies [9], [17], [18]. No external standard deviation was available so this was estimated by fitting a linear mixed model, with class, gender and their interactions as fixed effects as well as with cluster-specific random effects, to the baseline data. The estimated standard deviation (SD) was then calculated by summing the between- and within-cluster variances.

The Data Monitoring Committee for the CHAMPION Trial also had an oversight role for STRIPES.

The average cost per child in the intervention arms was calculated from total budget expenditures in Indian rupees, and the total number of children who sat the end trial test (table 1). To aid international comparison, costs were also converted to GBP and USD based on current rates (at 23^rd^ October 2012). We included only those children who sat the end trial test when measuring the total number of children who benefited from the programme.

**Table 1:** Costs per village and per child (Rupees, GBP, USD).

	Rupees	GBP*	USD*
***Fixed costs per village***			
Community volunteer and academic centre	22,810	264.82	424.49
Other fixed and overhead costs	36,578	424.68	680.71
Total fixed costs per village	59,388	689.50	1,105.21
***Variable costs per child***			
Material support per child given to all children, cost per child	200	2.32	3.72
Additional material support for girls, cost per child	1,400	16.25	26.05
***Average total costs per child who sat the end test***			
Average cost per tested child in a village not receiving girl child support	2,848	33.06	52.99
Average cost per tested child in a village receiving girl child support	3,628	42.12	67.52

* *Conversion date 23rd October 2012 (1 Indian rupee  = 0.01161 GBP/0.01861 USD).*

## Results

Figure 1a shows the number of villages and children through the various stages of the trial up to and including randomisation, and figure 1b shows the number of villages and children through the various stages of the trial and analysis. Data were available for all villages in the two intervention arms both at baseline and at the end of the trial. In the control arm, there were no data at baseline for one village, and no data at endline for another (different) village. Of the 16,581 children originally enumerated, 4,128 (25%) had a composite score from baseline testing, 4,378 (26%) had a composite score from endline testing, and 3,359 (20%) had a composite score from both baseline and endline testing. These percentages were similar in the three randomised groups (28% education intervention, 25% education intervention + kit for girls and 23% control; 30%, 26% and 25%; and 23%, 20% and 19% in the three groups respectively). The lower percentage in the control arm could reflect the loss of one cluster at each of the two time points.

Of the 4,029 girls originally enumerated in the two education intervention groups, 1151 (29%) had a composite score from baseline testing, 1,250 (31%) had a composite score from endline testing, and 973 (24%) had a composite score from both baseline and endline testing. These percentages were similar in the two randomised groups (31% education intervention, and 26% education intervention + kit at baseline; 32% and 30% at endline; and 26% and 22% at both base- and endline respectively).

Table 2 shows the baseline characteristics for the villages and the children. The villages were comparable in terms of their tribal status mean population size, as well as the numbers of teachers and blackboards per school.

**Table 2:** Baseline characteristics for clusters and children.

	Intervention 1 Education	Intervention 2 Education + kit for girls	Intervention All	Control
***Number of villages***	***53***	***54***	***107***	***107***
**Tribal status**				
non-tribal (n, %)	44, 83.0	47, 87.0	91, 85.0	94, 87.9
tribal (n, %)	9, 17.0	7, 13.0	16, 15.0	13, 12.1
**Size of village** (population mean, min, max)	1126, 160, 2496	1338, 220, 2499	1233, 160, 2499	1282, 160, 2498
**Number of teachers per school** * (mean, SD)	3.8, 2.1	4.2, 2.1	4.0, 2.1	4.1, 2.3
**Number of blackboards** * (mean, SD)	4.2, 2.2	4.3, 2.0	4.3, 2.1	4.3, 2.3
Enumerated children number (mean**, SD)	***4006 (76, 41)***	***4461 (83, 37)***	***8467 (79, 39)***	***8114 (76, 38)***
**Boys number** (%; mean**, SD)	2058 (51.4; 39, 22)	2380 (53.4; 44, 21)	4438 (52.4; 41, 22)	4110 (50.7; 38, 19)
**Girls number** (%; mean**, SD)	1948 (48.6; 37, 20)	2081 (46.6; 39, 17)	4029 (47.6; 38, 19)	4004 (49.3; 37, 20)
**Class 2: number** (%; mean**, SD)	1744 (43.5; 33, 19)	1821 (40.8; 34, 16)	3565 (42.1; 33, 17)	3297 (40.6; 31, 17)
**Class 3: number** (%; mean**, SD)	1201 (30.0; 23, 13)	1499 (33.6; 28, 13)	2700 (31.9; 25, 14)	2557 (31.5; 24, 12)
**Class 4: number** (%; mean**, SD)	1061 (26.5; 20, 12)	1141 (25.6; 21, 10)	2202 (26.0; 21, 11)	2260 (27.9; 21, 12)
**Baseline test- Boys and girls**				
composite score: number of children (mean**, SD)	1135 (21, 12)	1110 (21, 11)	2245 (21, 11)	1883 (18, 10)
maths: number of children (mean**, SD)	1138 (21, 12)	1114 (21, 11)	2252 (21, 11)	1888 (18, 10)
language: number of children (mean**, SD)	1135 (21,12)	1114 (21,11)	2249 (21,11)	1888 (18, 10)
Baseline composite score (mean, SD)	42.4, 21.2	44.0, 21.5	43.2, 21.4	41.4, 20.9
Baseline maths score (mean, SD)	38.8, 22.7	39.9, 22.8	39.3, 22.7	36.9, 22.0
Baseline language score (mean, SD)	45.9, 23.8	48.3, 23.9	47.1, 23.9	46.0, 23.7
**Baseline test- Boys**				
maths: number of boys (mean**, SD)	534 (10, 6)	561 (10, 6)	1095 (10, 6)	862 (8, 5)
composite score: number of boys (mean**, SD)	534 (10, 6)	560 (10, 6)	1094 (10, 6)	860 (8, 5)
language: number of boys (mean**, SD)	534 (10, 6)	561 (10, 6)	1095 (10, 6)	863 (8, 5)
Baseline composite score for boys (mean, SD)	42.7, 21.2	45.5, 21.5	44.2, 21.4	43.4, 20.7
Baseline maths score for boys (mean, SD)	39.4, 22.7	41.9, 23.2	40.7, 23.0	39.1, 22.0
Baseline language score for boys (mean, SD)	46.0, 24.1	49.1, 23.2	47.6, 23.7	47.6, 23.5
**Baseline test- Girls**				
composite score: number of girls (mean**, SD)	601 (11, 8)	550 (10,6)	1151 (11, 7)	1023 (10, 6)
maths: number of girls (mean**, SD)	604 (11, 8)	553 (10,6)	1157 (11, 7)	1026 (10, 6)
language: number of girls (mean**, SD)	601 (11, 8)	553 (10,6)	1154 (11, 7)	1025 (10, 6)
Baseline composite score for girls (mean, SD)	42.0, 21.3	42.5, 21.5	42.3, 21.4	39.8, 20.9
Baseline maths score for girls (mean, SD)	38.3, 22.6	37.8, 22.2	38.1, 22.4	35.0, 21.8
Baseline language score for girls (mean, SD)	45.8, 23.6	47.4, 24.6	46.5, 24.1	44.6, 23.9

* In main school in village (25 villages had 2 schools and 1 village had 3 schools). **Mean number per village.

**Figure 2:**
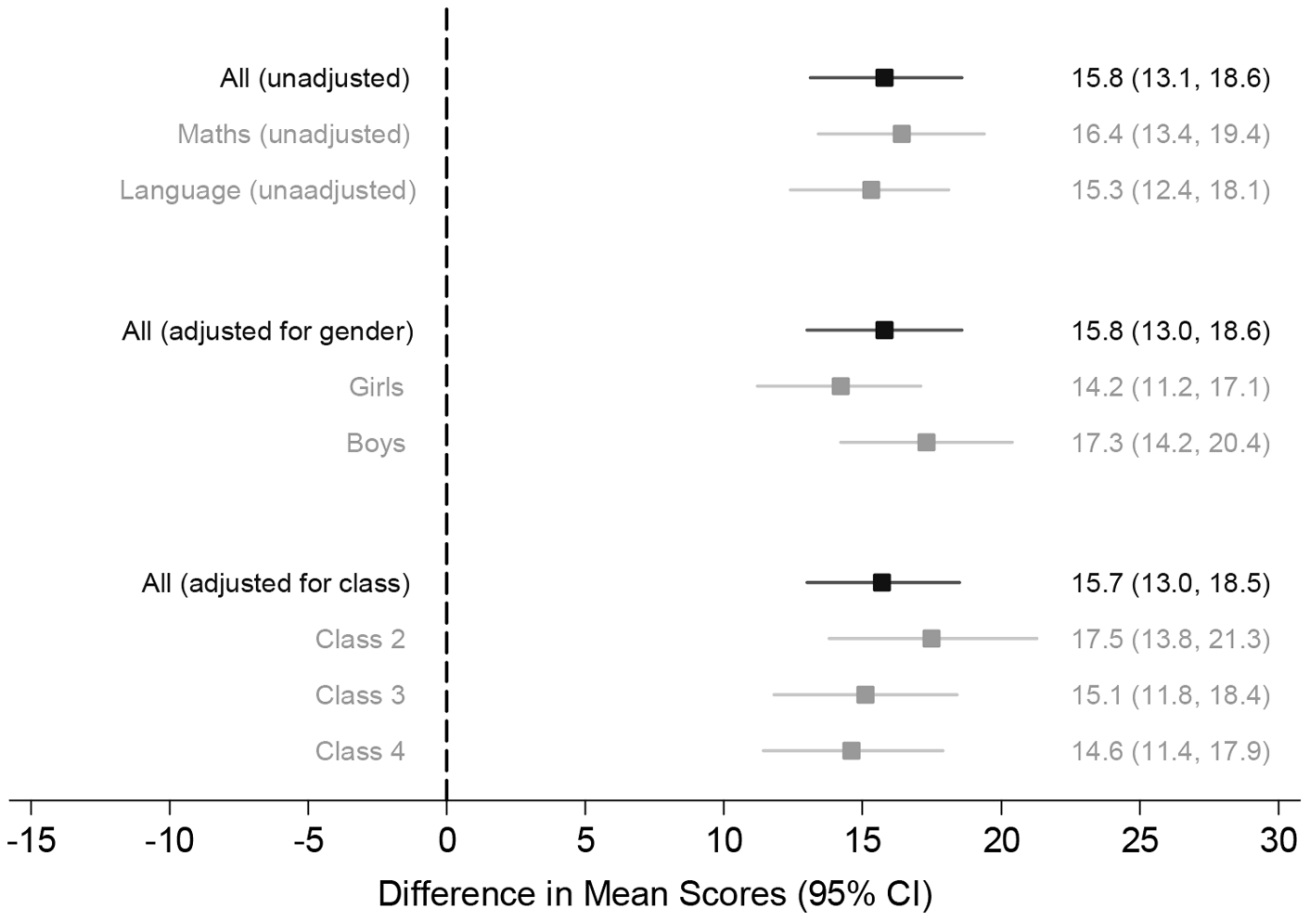
End of trial composite score: intervention vs control – overall and stratified by gender and baseline class.

Performance in the composite score as well as the maths and language test scores were largely similar at baseline, although there was some evidence that scores were slightly higher in the two intervention groups compared to the control group. This difference was greater for girls (a 2.5 point difference between the combined intervention group and the control group) than for boys (a 0.8 point difference).The SD of the baseline composite test score, estimated from the linear mixed model, was 21.2. Tables 3–4 and figure 2 show the results for comparison 1. Tables 5–6 show the results for comparison 2.

**Table 3:** End of trial composite scores in intervention vs. control villages.

	Educational intervention Number of villages = 107			Control Number of villages = 106*			Difference in means (95% CI; p-value)
	Number	Composite score		Number	Composite score		
	n	mean	SD	n	mean	SD	
**All children, unadjusted**	2364	60.2	19.3	2014	44.3	16.8	15.8, 13.1 to 18.6; <0.001
**All children, adjusted for gender**							15.8, 13.0 to 18.6; <0.001
**Stratified by gender** **							
**Boys**	1114	59.7	18.9	933	45.6	16.9	14.2, 11.2 to 17.1; <0.001
**Girls**	1250	60.5	19.7	1081	43.2	16.7	17.3, 14.2 to 20.4; <0.001
**All children, adjusted for class at start of intervention** **							15.7, 13.0 to 18.5; <0.001
**Stratified by class** §							
**Class 2**	808	62.2	19.5	654	44.7	17.7	17.5, 13.8 to 21.3; <0.001
**Class 3**	778	61.9	18.7	654	46.8	16.0	15.1, 11.8 to 18.4; <0.001
**Class 4**	778	56.3	19.1	706	41.6	16.3	14.6, 11.4 to 17.9; <0.001
**Children with baseline and end of trial score**	**Educational intervention number of villages = 107**			**Control number of villages = 105** †			
**All children, adjusted for baseline score and gender**	1829	60.8	19.3	1530	44.8	16.5	15.3, 12.8 to 17.8; <0.001
**Stratified by gender** ††							
**Boys**	856	60.3	18.8	696	45.6	16.6	14.4, 11.8 to 17.1; <0.001
**Girls**	973	61.2	19.8	834	44.2	16.4	16.0, 13.2 to 18.8; <0.001

* *In one village the test was not carried out at the end of the trial.*

** *Interaction between intervention and gender p = 0.008.*

§ *Interaction between intervention and baseline class p = 0.3.*

† *In one village test scores not available at baseline and in another village test scores not available at the end of the trial.*

†† *Interaction between intervention and gender after adjustment for baseline composite score p = 0.2.*

**Table 4:** End of trial maths and language scores in intervention vs. control villages.

	Educational intervention number of villages = 107			Control number of villages = 106*			Difference in means (95% CI; p-value)
	Number	Score		Number	Score		
	n	Mean	SD	N	Mean	SD	
**Final Maths Scores**							
**All children, unadjusted**	2372	55.3	22.3	2032	38.9	17.6	16.4, 13.4 to 19.4; <0.001
**All children, adjusted for gender**							16.4, 13.4 to 19.3; <0.001
**Stratified by gender** **							
**Boys**	1120	55.4	21.6	940	40.6	17.8	14.8, 11.7 to 17.9; <0.001
**Girls**	1252	55.2	22.8	1092	37.4	17.4	17.7, 14.4 to 21.0; <0.001
**Final Language Scores**							
**All children, unadjusted**	2370	65.0	19.3	2018	49.7	19.2	15.3, 12.4 to 18.1; <0.001
**All children, adjusted for gender**							15.3, 12.5 to 18.1; <0.001
**Stratified by gender** ***							
**Boys**	1118	63.9	19.4	935	50.5	19.0	13.4, 10.4 to 16.4; <0.001
**Girls**	1252	65.9	19.1	1083	49.0	19.3	16.9, 13.8 to 20.1; <0.001
	1839	56.0	22.3	1549	39.3	17.4	15.7, 13.0 to 18.4; <0.001
**Stratified by gender** §							
**Boys**	859	56.2	21.5	704	40.5	17.4	15.2, 12.4 to 18.1; <0.001
**Girls**	980	55.7	23.0	845	38.4	17.3	16.1, 13.1 to 19.2; <0.001
**Final Language Scores All children, adjusted for baseline score and gender**	1836	65.5	19.2	1535	50.4	18.8	14.9, 12.4 to 17.4; <0.001
**Stratified by gender** §§							
**Boys**	859	64.4	19.3	700	50.8	18.6	13.6, 10.9 to 16.3; <0.001
**Girls**	977	66.5	19.0	835	50.0	19.0	16.0, 13.1 to 18.8; <0.001

* *In one village the test was not carried out at the end of the trial.*

** *Interaction between intervention and gender p = 0.02.*

*** *Interaction between intervention and gender p = 0.008.*

§ *Interaction between intervention and gender for maths score after adjustment for baseline maths score p = 0.5.*

§§ *Interaction between intervention and gender for language score after adjustment for baseline language score p = 0.051.*

**Table 5:** End of trial composite score for educational interventions alone vs educational interventions + kits (girls only).

	Education intervention number of villages = 53			Education intervention + kit number of villages = 54			Difference in means (95% CI; p-value)
	number	composite score		number	composite score		
	n	Mean	SD	N	mean	SD	
**Girls**	636	60.3	19.9	614	60.8	19.4	0.5, −4.3 to 5.4; 0.8
**Girls, adjusted for class at start of intervention**							0.6, −4.3 to 5.5; 0.8
**Girls stratified by class at start of intervention** *							
Class 2	208	63.8	19.5	213	61.9	19.2	−1.9, −8.1 to 4.3; 0.5
Class 3	227	61.4	18.6	199	62.8	19.9	1.3, −4.5 to 7.1; 0.6
Class 4	201	55.3	20.9	202	57.8	18.9	2.5, −4.1 to 9.0; 0.5
**Girls adjusted for baseline score**	511	60.7	19.9	462	61.6	19.6	0.7, −3.6 to 5.0; 0.7

* *Interaction between intervention and class p = 0.4.*

**Table 6:** End of trial maths and language scores for educational interventions alone vs educational interventions + kits (girls only).

	Education intervention number of villages = 53			Education intervention + kit number of villages = 54			Difference in means (95% CI; p-value)
	Number	score		number	score		
	n	mean	SD	n	Mean	SD	
**Girls**							
**Final Maths Score**	637	54.4	23.2	615	55.9	22.4	1.6, −3.9 to 7.1; 0.6
**Final Language Score**	637	66.1	19.2	615	65.7	19.1	−0.4, −4.9 to 4.0; 0.8
**Girls adjusted for baseline scores**							
**Final Maths Score**	514	54.7	23.6	466	56.9	22.4	2.5, −2.5 to 7.5; 0.3
**Final Language Score**	512	66.7	18.7	465	66.3	19.3	−0.9, −4.9 to 3.1; 0.7

Children from villages in the educational intervention groups had significantly higher composite test scores than in control villages at the end of the trial, and this difference was statistically significant (mean difference 15.8; 95% CI 13.1 to 18.6; p<0.001) (table 3 and figure 2). This effect appeared larger for girls than boys (p-value for test of interaction between intervention and gender  = 0.008). The benefits of intervention were consistent across the three classes, two, three, and four (p-value for test of interaction between intervention and class  = 0.3) (table 3 and figure 2). Table 3 also shows the effect of intervention on the primary outcome after adjustment for scores at baseline. There was similar benefit of intervention as without baseline adjustment (mean difference 15.3; 95% CI 12.8 to 17.8; p<0.001) for all children. However, the test for interaction between intervention and gender was no longer statistically significant (p-value for interaction  = 0.2). Using the SD of the composite score at baseline, the mean difference of 15.8 in percentage score translates into a 0.75 SD difference.

Similar benefits of the intervention were seen for the secondary outcomes of individual maths and language test scores both for all children and for boys and girls separately. This effect appeared larger for girls than boys (p-value for test of interaction between intervention and gender 0.02 for maths, and 0.008 for language) although as with the composite score, differences between intervention and control were less marked and no longer statistically significant after adjustment for baseline scores (table 4).

For comparison 2, i.e. the effect of providing the materials kit to girls, we estimate a 0.5 percentage point increase in composite test scores at the end of the trial relative to the scores of girls in villages which did not receive kits. This difference is not statistically significant. (95% CI -4.3 to 5.4; p = 0.8, see table 5). The lack of detectable benefit for the additional materials for girls intervention was consistent across the three classes (p-value for test of interaction between intervention and Class  = 0.4). Table 5 also shows the effect of intervention on the primary outcome, after adjusting for the scores on the baseline test for those girls who had both baseline and endline scores. Again, there is no evidence of benefit of intervention (mean difference 0.7; 95% CI -3.6 to 5.0; p = 0.7).

This finding, a lack of detectable benefits of the materials and teaching intervention relative to only supplementary teaching was seen for both secondary outcomes; maths and language test scores separately and for analyses in which baseline test scores were taken into account (Table 6).

The average cost per child for the two year intervention was Rs.2,848 (£33.06, $52.99) for villages which did not receive the additional material support, and Rs.3,628 (£42.12, $67.52) for villages which did receive additional material support. This is equivalent to a cost of Rs.382.97 (£4.45, $7.13) per 0.1 SD increase in composite test score for the intervention without kits, and Rs.480.59 (£5.58, $8.94) for the intervention with kits. These costs are calculated using the total number of children who sat the endline test.

## Discussion

Two-hour after-school instruction classes led by a trained community volunteer in a large cluster randomised trial significantly improved the composite, maths and language scores in government primary schools in rural Andhra Pradesh. Both girls and boys in the intervention groups did better than their counterparts in control groups. In contrast, girls who received additional material support along with the after school instruction did not achieve better scores than girls who did receive supplementary instruction but not the additional material support.

Two important methodological strengths of the study are its large size and its rigorous randomised design. In particular, there are two major background characteristics, parents' economic status and education levels, which could influence outcomes but randomisation should have distributed these potentially confounding characteristics evenly between the groups.

Our study did not find a notable difference between the performance of girls who did and did not receive the kit of supplementary materials. This is in line with earlier studies evaluating the impact of providing only material support to children which found that it has minimal or no impact on learning levels [19], [20].

This study has a few key limitations. It was not possible to blind participants or to ensure that outcome assessors were blind.

Secondly, we do not know if the effect of the intervention persisted after the intervention was completed as our study did not continue to conduct further follow up. Evidence from similar studies suggests that measured effects do often persist well after the intervention ceases [9], [21].

In addition, we did not collect data about other outcomes such as school attendance therefore cannot assess whether the interventions (especially the kits for girls) had effects other than on maths and language scores. Another limitation of our approach is that we require that CVs be relatively highly-educated (10^th^ class where possible). In scaling the intervention up to other disadvantaged settings this may be a major constraint.

The proportions of children attending the baseline test who also attended the test at the end of the trial were reasonably high (81.5% (1829 of 2245) for the combined intervention groups and 81.3% (1530 of 1883) for the control group), an attrition percentage which compares favourably to other education studies in India [9], [10]. However the proportions of enumerated children who performed the tests are low: our primary analysis of composite test scores at the end of the trial includes only 27.9% (2364 of 8467) and 24.8% (2014 of 8114) of children enumerated for the (combined) intervention and control groups respectively. There are a number of factors which contribute to these low percentages. First there was a gap between enumeration and the baseline test, with the latter taking place at a time when there was little agricultural work available and therefore high out-migration. Second some children went to school outside their villages (e.g. to private schools) and were not present in villages on the day of the tests but we had informed them about the tests and encouraged them to attend the tests. Third the researchers collecting the enumeration data were told to include all potentially eligible children in each household in order to be sure to capture any child that was eligible at trial start. The numbers may have been inflated by inclusion of temporary migrants that parents reported might be in the village at the start of the year, and children whose ages and grade standards in the following year could not be verified during the short enumeration visits.

Estimating the impact of attrition both in the period prior to the baseline test and between the baseline and follow-up tests is, of necessity, speculative. Considering first attrition between the two tests, the proportions not attending the follow-up tests are almost identical in the control and intervention groups, providing no evidence that reasons for non-attendance at the follow-up test might differ markedly between the randomised groups. We have no evidence, for example, to suggest that attrition between the two tests in the control group reflects the fact that such children were receiving additional education whilst those in the intervention were not. In our view it is most plausible that the non-attenders in the intervention group will have received some benefit, but not as much as those who attended the test; whilst non-attenders in the control group are unlikely to have done better than those who attended the test had they actually done so. For these reasons we judge an assumption that mean test scores among those who did not return for the second test would be the same in the randomised groups as likely to be conservative. Making such an assumption reduces the estimated impact of the intervention by 18.6% (the attrition rate in the groups as a whole); from 0.75 SD to 0.61 SD. We believe that this can be considered a realistic lower bound on our estimate of the effectiveness of the intervention amongst those taking the baseline test.

Turning now to attrition prior to the baseline test, we have no evidence that those who attended the tests were unrepresentative of children who were enumerated and again the fact that attrition rates are similar in the control and intervention groups provides no evidence that reasons for the attrition differs markedly between the randomised groups. In our view extrapolating to the whole enumerated population has limited utility since, were the intervention implemented widely, those migrating would also be accessing the intervention irrespective of where they were resident. However if one did wish to extrapolate to the whole enumerated population, again making the assumption that mean follow-up scores in those who did not attend did not differ between the two groups, the estimated impact of the intervention would be reduced by 73.6% (the attrition rate in the groups as a whole), from 0.75 SD to 0.20 SD.

In some ways, the supplementary remedial instruction in our study is similar to other programmes using low-cost para-teachers introduced by several state governments in India since the mid-1990 s [7]. However, our primary treatment effect estimate is a 0.75 SD improvement in scores, which is large relative to other studies evaluating educational interventions across the developing world. Two similar interventions run in India registered a 0.28 SD improvement and a non-significant difference, respectively [9], [22]. A recent review catalogues trials which found treatment effect estimates between a 0.15 and 0.3 SD improvements in test scores for educational interventions in needy areas [8].

Our large treatment effect may reflect the ways the STRIPES interventions differ from previously attempted interventions. There was rigorous monitoring of the CV by the trial team to help address absenteeism. This included drop-in observations by team members, which were conducted twice each week, and monthly review meetings between CVs and the trial team. Teacher absenteeism is a major problem in India. A study [5] which included unannounced visits to a nationally representative sample of government primary schools in India found 25% of teachers were absent, and only about half were teaching, We speculate monitoring by the STRIPES intervention team led to an increase in time spent on learning as there was a consistent availability of a teacher.

The STRIPES intervention used supplementary teaching-learning material based on grade-specific, local state curriculum in the form of workbooks for children and teachers. This was different to similar studies [9] where the materials were developed based on a standardised curriculum developed by the intervention team. The monthly reviews included a detailed appraisal of children's progress at the ASC and training on any gaps in learning that were picked up. A dedicated expert in pedagogy from Naandi's Educational Resource Group was also part of the trial design team and ensured that the concepts were being taught in the correct manner.

The monthly parent-trial team-school teacher meetings emphasised the value of education and strengthened the ties between parents, children, and teachers. This is consistent with the results of an evaluation [22] which found that villages where local community members were trained to hold remedial reading camps, there was community participation and improved educational outcomes especially in teaching illiterate children to begin to read.

The large magnitude of our treatment effect estimates may be partly because most previous studies evaluated pilot interventions which were almost certainly subject to “growing pains” and the process of learning from mistakes. Other work suggests this may underestimate true treatment effects of such programmes [23].

Additionally, it may be possible that our intervention teachers were teaching only to the test. Previous studies have documented that such teaching to the test has fewer long term benefits [9]. To minimize teaching to the test, the TLM developed by Naandi were based on the national curriculum. The CRL pedagogy ensured that CVs focused using the TLM to teach by promoting social interaction and peer-learning. Diverse exercises and activities focused on the steps, purpose and the context in which computations were to be done rather than on getting the ‘right’ answer. Therefore, the focus was on learning rather than on answers to a question. Indeed, our treatment effect estimates are large enough to suggest that substantial learning did occur. In addition, we attempted to minimise a possible bias related to designing the evaluation instrument as Educational Initiatives, had worked with the Naandi foundation previously and, so possibly knowledge of the type of instruments used by Educational Initiatives may have filtered down to the CV level. However, there was no overlap between the test designers and test administrators (GH Consultancy Ltd), and Naandi Foundation workers were not at any of the test sites on the day of the test.

Finally, our intervention took place in an area which is particularly needy and thus has more to gain from the intervention than previous study sites might have had. Initial learning levels were lower at baseline in our trial area than in other areas where such evaluations were implemented. This suggests larger gains to be made with relatively simple interventions when initial levels are lower and, in turn larger potential treatment effects. Similar results have been found in evaluations of primary education interventions in other particularly needy areas such as rural Afghanistan [24].

We know of no comparable published studies measuring cost effectiveness of educational interventions in rural India. One study in urban India, where the reported test score improvements were substantially lower than those reported in this study, found total costs of $4.50 per child over a two year period [9]. Their cost estimate included only the cost of additional teachers, and did not appear to include costs for additional supervision, training, hiring, and related infrastructure needed to implement these programmes. We believe projects in remote, rural regions will be substantially more costly than urban projects due to greater logistical issues including transport and supervision costs. We have also measured costs conservatively, as we assume only those children who completed end-line tests benefitted from the project. Average costs per child would be substantially lower if we assumed all children enumerated in the village benefitted equally from the intervention.

The study took place in largely remote area and villages underserved by the government educational apparatus. It is likely that the findings of our study are generalisable to similar areas which abound in rural parts of the developing world.

## Conclusion

The STRIPES trial corroborates the few other studies which find that supplementary remedial education programmes can have a large positive impact on learning levels [8], [9]. It provides some of the first evidence that this type of intervention can be implemented in remote rural areas which are underserved by the government and still have a large effect and also provides evidence that longstanding NGO interventions may be more effective than interventions tailor-made for academic studies. The results of this paper could be applied to numerous other settings, in India and beyond, which closely resemble our trial area in terms of size, remoteness and level of services provided by the government.

## Supporting Information

Checklist S1 **CONSORT checklist for the trial.**
(DOCX)

Checklist S2 **CONSORT checklist for the trial.**
(DOCX)

Protocol S1 **Protocol for the STRIPES trial.**
(PDF)

Box S1 **Further details of intervention.**
(DOCX)
