# Correction: The Support to Rural India's Public Education System (STRIPES) Trial: A Cluster Randomised Controlled Trial of Supplementary Teaching, Learning Material and Material Support

**DOI:** 10.1371/annotation/75418564-edc5-465e-b94b-1ee3b8cf39e5

**Published:** 2014-01-23

**Authors:** Rashmi Lakshminarayana, Alex Eble, Preetha Bhakta, Chris Frost, Peter Boone, Diana Elbourne, Vera Mann

In Figure 1B, the figures for girls and boys in the primary analysis should be reversed. Please see the corrected version of Figure 1B here: 

**Figure:**
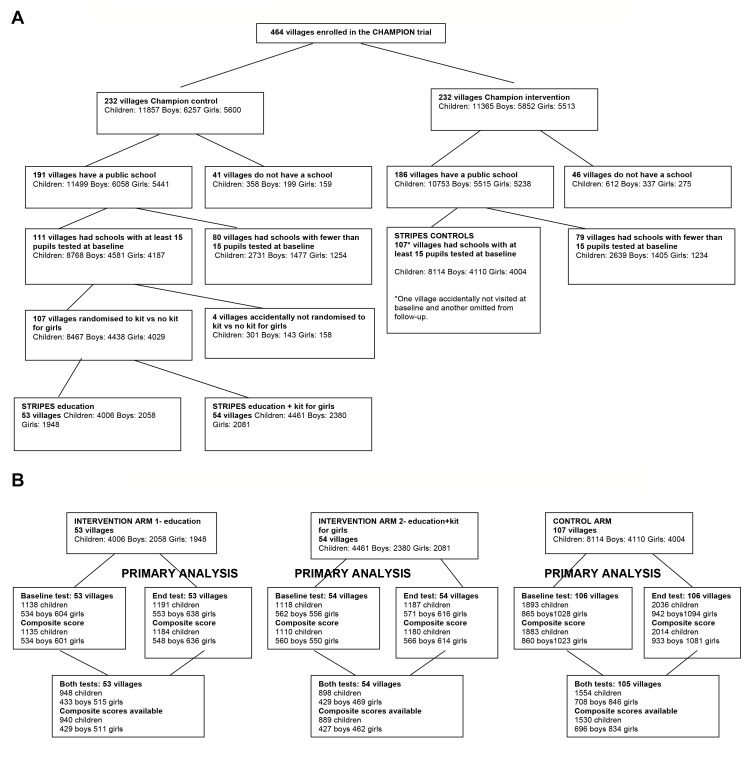


There were multiple errors in the author contributions. The updated author contributions are as follows:

Conceived and designed the experiments: AE PBo DE VM CF RL PBh.

Performed the experiments: RL PBh. 

Analyzed the data: VM CF PBo AE DE. 

Contributed reagents/materials/analysis tools: RL AE PBh DE CF PBo VM. 

Wrote the paper: RL AE DE CF PBo VM PBh.

