# Supplementary material for: The Support to Rural India's Public Education System (STRIPES) Trial: A Cluster Randomised Controlled Trial of Supplementary Teaching, Learning Material and Material Support
Source: PLoS One. 2013 Jul 16;8(7):e65775. doi: 10.1371/journal.pone.0065775 (PMC3712986; doi:10.1371/journal.pone.0065775)
Supplement: Checklist S2 — CONSORT checklist for the trial. (DOCX) [file pone.0065775.s002.docx]

**Checklist S2: Extension of CONSORT for abstracts in cluster randomised trials**

| **Item** | **Standard Checklist item** | **Extension for cluster trials** | **Reported on line number** |
| --- | --- | --- | --- |
| **Title** | Identification of study as randomised | Identification of study as cluster randomised | **1-3** |
|  |  |  |  |
| **Trial design** | Description of the trial design (e.g. parallel, cluster, non-inferiority) |  | **8-9** |
| **Methods** |  |  |  |
| **Participants** | Eligibility criteria for participants and the settings where the data were collected | Eligibility criteria for clusters | **5-6, 9-11** |
| **Interventions** | Interventions intended for each group |  | **3-4** |
| **Objective** | Specific objective or hypothesis | Whether objective or hypothesis pertains to the cluster level, the individual participant level or both | **2-6** |
| **Outcome** | Clearly defined primary outcome for this report | Whether the primary outcome pertains to the cluster level, the individual participant level or both | **4-6** |
| **Randomization** | How participants were allocated to interventions | How clusters were allocated to interventions | **8-11** |
| **Blinding (masking)** | Whether or not participants, care givers, and those assessing the outcomes were blinded to group assignment |  | **11-12** |
| **Results** |  |  |  |
| **Numbers randomized** | Number of participants randomized to each group | Number of clusters randomized to each group | **8-11, 14-18** |
|  |  |  |  |
| **Numbers analysed** | Number of participants analysed in each group | Number of clusters analysed in each group | **8-11, 14-18** |
| **Outcome** | For the primary outcome, a result for each group and the estimated effect size and its precision | Results at the cluster or individual participant level as applicable for each primary outcome | **14-19** |
| **Harms** | Important adverse events |  | **None** |
| **Conclusions** | General interpretation of the results |  | **22-25** |
| **Trial registration** | Registration number and name of trial register |  | **26-27** |
| **Funding** | Source of funding |  | **28-30** |
|  |  |  |  |
