# Supplementary material for: The Support to Rural India's Public Education System (STRIPES) Trial: A Cluster Randomised Controlled Trial of Supplementary Teaching, Learning Material and Material Support
Source: PLoS One. 2013 Jul 16;8(7):e65775. doi: 10.1371/journal.pone.0065775 (PMC3712986; doi:10.1371/journal.pone.0065775)
Supplement: Protocol S1 — Protocol for the STRIPES trial. (PDF) [file pone.0065775.s003.pdf]

Eble et al. Trials 2010, 11:10
http://www.trialsjournal.com/content/11/1/10

TRIALS

STUDY PROTOCOL Open Access

The STRIPES Trial - Support to Rural India's Public Education System

Alex Eble[1], Vera Mann[2*], Preetha Bhakta[3], Rashmi Lakshminarayana[1], Chris Frost[2], Diana Elbourne[2], Peter Boone[1]

Abstract

Background: Performance of primary school students in India lags far below government expectations, and major disparity exists between rural and urban areas. The Naandi Foundation has designed and implemented a programme using community members to deliver after-school academic support for children in over 1,100 schools in five Indian states. Assessments to date suggest that it might have a substantial effect. This trial aims to evaluate the impact of this programme in villages of rural Andhra Pradesh and will compare test scores for children in three arms: a control and two intervention arms. In both intervention arms additional after-school instruction and learning materials will be offered to all eligible children and in one arm girls will also receive an additional 'kit' with a uniform and clothes.

Methods/Design: The trial is a cluster-randomised controlled trial conducted in conjunction with the CHAMPION trial. In the CHAMPION trial 464 villages were randomised so that half receive health interventions aiming to reduce neonatal mortality. STRIPES will be introduced in those CHAMPION villages which have a public primary school attended by at least 15 students at the time of a baseline test in 2008. 214 villages of the 464 were found to fulfil above criteria, 107 belonging to the control and 107 to the intervention arm of the CHAMPION trial. These latter 107 villages will serve as control villages in the STRIPES trial. A further randomisation will be carried out within the 107 STRIPES intervention villages allocating half to receive an additional kit for girls on the top of the instruction and learning materials. The primary outcome of the trial is a composite maths and language test score.

Discussion: The study is designed to measure (i) whether the educational intervention affects the exam score of children compared to the control arm, (ii) if the exam scores of girls who receive the additional kit are different from those of girls living in the other STRIPES intervention arm. One of the goals of the STRIPES trial is to provide benefit to the controls of the CHAMPION trial. We will also conduct a cost-benefit analysis in which we calculate the programme cost for 0.1 standard deviation improvement for both intervention arms.

Trial Registration: Current controlled trials ISRCTN69951502

Background

Education in India

Effective delivery of primary education, particularly to girls, is a priority across the developing world and certainly to the Indian government. India's 86th Constitutional Amendment Act, passed in December 2002, mandated that free and compulsory education for all children between six and 14 years of age should be a fundamental right protected by the government and its achievement a high priority [1]. 'Sarva Shiksha Abhiyan' (abbreviated as SSA, also known as the 'Education for All' movement), a flagship programme of the Government of India, was created to achieve universal elementary education in the country, as mandated by the aforementioned constitutional amendment. The programme aims to achieve the goal of universal elementary education of satisfactory quality by 2010 [2].

Unfortunately, the programme is falling short of its mandate. One of the goals of SSA is that all children complete eight years of schooling. The Annual Status of Education Report (ASER), performed by Pratham, an Indian NGO, recorded in 2007 that 13.5% of 14 year-old children in India are not in school and an additional 14.4% of those that are in school and should be finishing the eighth standard are studying in standard six or lower

* Correspondence: vera.mann@lshtm.ac.uk
[2]The London School of Hygiene and Tropical Medicine, Keppel Street, London WC1E 7HT, UK

© 2010 Eble et al; licensee BioMed Central Ltd. This is an Open Access article distributed under the terms of the Creative Commons Attribution License (http://creativecommons.org/licenses/by/2.0), which permits unrestricted use, distribution, and reproduction in any medium, provided the original work is properly cited.

[3]. This implies that over 25% of the children who were 10 years old when the constitution was amended in 2002 did not complete elementary education as mandated by the amendment.

Some indicators of educational provision in India encourage optimism. The national enrolment rate among six to 14 year olds, for example, is 93.5 percent [3]. The enrolment rate for Indian children between the ages of seven and ten is more than 95 percent. Unfortunately, enrolment does not equate with performance. Evidence suggests the overall quality of the education delivered by the Indian government is still not optimal. More than 33% of Indian children enrolled in standards three to five are unable to read the assigned text for the first standard, and even more are unable to perform simple subtraction [3]. India's country-wide adult literacy rate is only 55%, and the adult literacy rate for women is 45% [4]. In response to the perception that the quality of public education is not optimal, 20% of children now attend private schools [5].

The situation in Andhra Pradesh

Despite strong economic performance, the state of Andhra Pradesh (AP) ranks near the bottom of the list of 28 Indian states using several country-wide education metrics. While ranking 11th out of 28 in terms of per capita State Domestic Product (SDP), AP ranks 22nd of 28 in terms of adult literacy. Drop-out rates are also high in AP. The STRIPES trial will take place in the Telangana region of AP, home to the highest drop out rates (46%) in primary schools in the State [6]. Educational attainment in the region is notably low. In the Mahabubnagar district of AP, only 52.5% of children in Standards 3 to 5 are able to read text assigned in the first standard and only 64.2% are able to perform simple subtraction, both of which are competencies expected of them before entering these grade levels [3].

It is also important to note that boys often receive preferential treatment compared to girls at school and within the household. In the country-wide assessment cited above, girls performed consistently below boys in competency indicators and educational attainment. Numerous studies document that girls regularly underperform relative to boys. There is also a robust literature documenting that education of girls has potential long term benefits. Educated girls become better educated women who raise healthier, better-educated children and have improved household financial management. These benefits do not accrue with the education of boys [7-11].

The Naandi programme

Over the past five years, the Naandi Foundation has been working towards ensuring that every underprivileged child gets academic and social support so that he or she completes 10 years of schooling. One intervention stemming from this mission is a programme Naandi has developed in rural and urban areas to provide material support alongside after-school instruction to underprivileged children (see the Interventions section below). There is some evidence that programmes such as this could significantly improve academic performance, attendance and retention of children. The Naandi Foundation has conducted unpublished independent assessments of their programmes by comparing children's performance on tests at the end of the programme to baseline tests. These assessments suggest children do benefit from the programme. A similar programme in an urban setting (Vadodara and Mumbai) provided remedial education to students who were falling behind in school. The assessment of the project measured 0.14 and 0.28 standard deviation increases in average test scores in the intervention arm when compared to controls during the first and second years, respectively [12].

In the STRIPES intervention, a member of the recipient community is trained to serve as an instructor for a daily after-school study session. The accompanying programme management team engages in an extensive outreach programme, not only to involve the recipient community in selection of the teacher, but also to promote education as a common value in project areas. Evidence suggests that such community-focused interventions may be effective. A randomised experiment evaluating a programme in Uganda promoting community monitoring of health service delivery found that the programme resulted in more services being delivered and improved health outcomes [13]. An evaluation of another programme in El Salvador that focused on community management of schools suggested the programme was responsible for increased learning levels of students [14].

Similarly, there is some evidence that providing material support to families for children's education may increase retention rates. Another intervention run as a randomised controlled experiment provided materials to school children in Kenya and found promising results to support the claim of a link between material provision and increased retention rates. In the study, an NGO-led intervention distributed textbooks to a set of schools in Kenya and the effects of the intervention were evaluated in relation to a control group. The study found that dropout rates "fell considerably in treatment schools, and in five years pupils in treatment schools completed about 15 percent more schooling" [15].

Aims of the trial

The STRIPES trial will add to understanding about the effectiveness of programmes to support education in rural settings in AP and similar areas. The trial will assess whether a programme providing additional

educational instruction and learning materials, when compared to usual government provision of education, is effective. It will also assess whether provision of a supplementary kit of materials (a school uniform, bag and other materials) to girls in addition to this programme is more effective than the programme alone. This question is particularly relevant given the expense of providing such kits to children and the widespread use of these kits by charity organisations.

Both interventions are based on the Ensuring Children Learn and Nanhi Kali programmes run by the Naandi Foundation in five Indian states. The results of this trial will be used to help determine expansion plans for this programme.

The study is conducted in conjunction with the CHAMPION trial and one of its major goals is to provide benefits to the controls of the CHAMPION trial.

Methods/Design

This study is a cluster-randomised controlled trial involving 214 villages and public primary schools in the Nagarkurnool district, with the village as the primary unit of randomisation. This is an unblinded study as, following randomisation, participants will be aware of whether or not they are in an intervention or control village.

Endpoints of the study

The primary endpoint of this study is a composite of scores earned on language and mathematics assessments from an 'endline' test which will be conducted at the end of the programme.

Secondary endpoints to be analysed will include:

◦ Scores on language and maths assessments, separately
◦ Cost-benefit ratios for the two interventions.

Further information on the theory and methods behind the collection of test data, as well as collection of other secondary endpoints, is given in the "Data collection" section.

Trial Population

In January 2008 an enumeration team used a baseline survey to collect data from all children aged between 4 and 12 in each of the 464 CHAMPION villages. Although the STRIPES education intervention will be offered to all children attending schools in the eligible STRIPES intervention villages, only enumerated children will be included in the statistical analysis of the trial.

Randomisation

This study is conducted in conjunction with the CHAMPION Trial [16]. Due to this, the randomisation will be conducted in two stages.

In stage one, randomisation for the CHAMPION trial, 464 villages in the Mahabubnagar district were randomised to receive either a health intervention (and therefore to serve as education trial controls) or an education intervention (and therefore to serve as health trial controls). This randomisation was stratified by distance of the village to the nearest designated secondary healthcare facilities and by the tribal status of the village. 232 villages were allocated to receive the health intervention and 232 were allocated to receive the education intervention. Of these 464 villages, there were 376 villages with at least one primary public school (operating in the 2007-8 academic year and intending to operate for the duration of the trial). In these 376 villages, baseline testing was carried out for all students present in the village on the day of testing. Among these villages, only 214 had 15 or more children available for testing, the minimum number of students to make the intervention cost-effective. Only these 214 villages are included in the STRIPES trial. The remaining villages allocated to receive the education intervention but ineligible for the trial have been offered the same material support programme as trial intervention villages and invited to attend the after-school tuition programme in the nearest intervention school.

In stage two, the education interventions will be randomly allocated amongst the 107 villages allocated to receive the education intervention in the first randomisation that were eligible in the criteria outlined above. These villages will be randomised to either receive supplementary teaching plus learning materials or supplementary teaching plus learning materials and, for girls only, additional material support which includes two uniforms, two pairs of undergarments, a pair of sandals and a school bag. Every public primary school in these villages will be a base for the intervention. Eligible children living in these villages will be invited to receive the intervention. Eligible villages assigned to receive the health intervention will serve as controls for the STRIPES trial. The flowchart of the randomisation is shown in figure 1.

STRIPES Intervention groups

In intervention villages, we will deliver one of the following two packages of interventions:

1. Supplementary teaching + learning material

For each eligible school, the trial will identify a "Community Activist" (henceforth "CA") who will be trained to deliver supplementary lessons to all children in standards 2, 3 and 4 for year 1 and in standards 3, 4 and 5 in year 2 of the trial. In each village, the intervention will begin with a community meeting in which all parents are brought together to suggest potential CAs for interview and recruitment. The CAs will then be trained by the Naandi Education Research Group team.

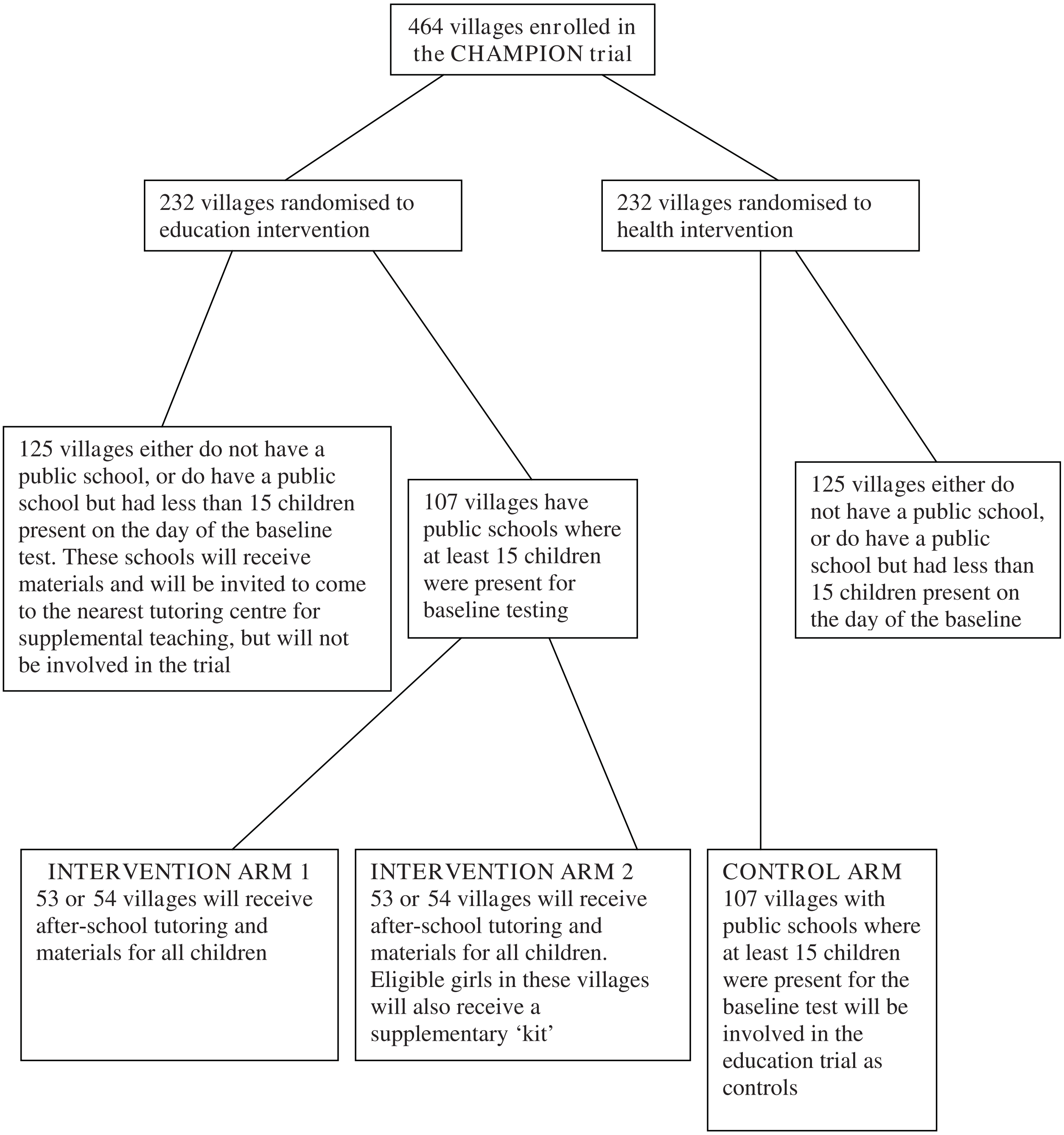

Figure 1 Flowchart of the STRIPES randomisation.

The CA then takes responsibility for ensuring children's attendance at these lessons through engaging families of eligible children. The family will enter an oral agreement with the CA that they will ensure that their children attend. This process of community involvement is intended to galvanise families to take responsibility for their children's attendance and performance in school.

After this initial period of introduction and community sensitisation, the CA will commence providing remedial instruction, in schools if possible, after normal school hours on a daily basis. The subject matter covered in these sessions will reinforce the curriculum covered in school and will be tailored to students' grade-specific needs and learning levels. The material to be used in the lessons has been developed and tested by

education experts from both the Naandi Foundation and external consultants and has been used extensively in the Naandi Foundation's education programmes over the last four years. A bundle of learning materials, including a pen, four pencils, two notebooks, a ruler and an eraser, will be provided to each participating child for use in these supplementary classes.

2. Supplementary teaching and learning material plus, for girls only, additional material support

For each eligible village in this group, the trial will provide the services outlined above and will also provide each girl student with a kit of materials intended to improve her attendance and, through increased attendance, performance in school. The kit includes a uniform, shoes, socks, undergarments and a school bag. As detailed in the background section, this aspect of the intervention is provided primarily with the goal of increasing the likelihood that girls attend and stay in school. The logic of this intervention is that increased attendance translates to increased exposure to the material taught in school and through this exposure improved performance on tests. Furthermore, girls face greater obstacles in attaining education than boys and educating girls has greater leverage on generating income, improving productivity and reducing family size than does educating boys [11].

STRIPES Control group

In the control group no education programme will be implemented. The health interventions offered to these villages are discussed in more detail in the CHAMPION Trial Protocol [16]. The programme will involve community education for mothers, safe home deliveries and contracting out to the private sector for complicated deliveries that cannot be safely handled at home. It is anticipated that these interventions in the STRIPES control group will have negligible impact on academic performance of children enrolled in or eligible for the second, third and fourth standard at the start of the trial.

Duration

This programme is scheduled to run the course of two full school years, starting in October 2008 and ending by April 2010 with the final assessment.

Trial site(s) and eligible schools

The trial site is the Nagarkurnool division of Mahabubnagar district in the Indian state of Andhra Pradesh. Mahabubnagar is rated as one of the districts in Andhra Pradesh with the highest proportion of children out of school in the state [3]. The trial will involve only those villages in the division with a population of less than 2,500 people already in the CHAMPION trial. This criterion was decided upon in light of the intervention targeting neonatal mortality that takes place in control villages. Under this criterion, a total of 464 villages were initially potentially eligible for the trial, with a total population of approximately 300,000 people.

Additionally, an eligible village must have at least one public primary school serving boys and girls. The school must be operating in the 2007-2008 academic year and be likely to continue operations during the following two years. There must have been at least 15 children in standards 2, 3 and 4 in the school present on the day of baseline testing conducted in the summer and autumn of 2008. Only enumerated children residing in an eligible village and attending the 2nd, 3rd or 4th standard in the government school in their village of enumeration during the 2008-9 school year are eligible for enrolment in the trial.

To maintain the integrity of the data collected in the CHAMPION trial and to ensure all participants in the CHAMPION and STRIPES trials receive an intervention, those villages which are assigned to receive the STRIPES intervention (i.e. CHAMPION controls) but not eligible for enrolment in the STRIPES trial, because they either lack a school or a critical mass of students, will receive the same materials distributed to all children and will be invited to participate in the tuition programme occurring in the nearest STRIPES intervention village.

Eligibile children

A child is eligible for inclusion in the analysis of the trial if s/he satisfies the following criteria:

- S/he is resident in an eligible village
- S/he has been recorded in the enumeration in January 2008 as planning to be enrolled in the 2nd, 3rd or 4th standard at the government school located in her/his village in the in 2008-9 academic year.
- After hearing an explanation of the trial, her/his parent or guardian did not choose to opt out

Eligibility for primary analysis

The primary analysis will be conducted according to intention to treat as determined in the initial randomisation. All children enrolled in the trial satisfying eligibility criteria will be included in the primary analysis. The primary analysis will consist of:

- All STRIPES intervention children compared to all STRIPES control children
- All STRIPES intervention girls allocated a kit compared to all STRIPES intervention girls NOT allocated a kit

Enumerating children

Prior to randomisation, enumeration was carried out in all villages collecting data on the eligible children residing in each household through household surveys.

Dealing with migration

The population of the villages eligible for the trial will be dynamic due to migration (largely temporary for seasonal labour). If migrants take their children with them, this may dilute the effect of the supplementary teaching on test performance and will obviously influence the attendance of the children in question. We will not attempt to follow-up eligible children who migrate. If they return to their village during assessment periods they will be invited with all the other eligible children to do the test. We will not include in the analysis results for children of families who migrate into the village after the initial enumeration.

Losses to follow-up

Data is collected on each child at the school in her/his enumeration village. The main challenge is motivating the children to take the test, particularly those children not receiving the education intervention. To address this problem, we provide all test takers with a small packet, which includes pencil, sharpener, eraser, ruler and notebook, as an incentive to take the test.

Consent

The consent process in this trial involves the state, Panchayat, schools, parents and children. At the state level, approval of the protocol has been obtained from the Department of Education of the Government of Andhra Pradesh. We have obtained written consent from Panchayat leaders to conduct the CHAMPION and STRIPES trials in the villages in their respective Panchayats. The Panchayat is a democratically elected body that governs a small group of villages. This is the smallest unit of government in rural India. In all villages in the trial, consent was obtained from the Panchayat during the Gram Sabha, a meeting of citizens belonging to a given Panchayat. Members of the CHAMPION Trial research team explained to each Panchayat the two interventions, health and education, the process of randomisation, and what participating for the trial entailed for the Panchayat. The villagers discussed this and gave consent both orally and in writing through the signature of the Panchayat leader. This process of obtaining consent through meetings with approval of the 'guardians' of the clusters is common in trials in which the intervention is delivered at the level of a cluster and it is not possible to obtain informed consent for randomisation from individuals within the cluster [17,18]. We then obtained further consent to randomise between the two alternative education interventions from the Panchayats of villages assigned to receive the STRIPES education intervention.

Members of the intervention team informed parents or guardians of children about the trial in both STRIPES intervention arms prior to delivery of the intervention. We discussed all aspects of the trial with them and explained that they had the opportunity to opt out of the trial. If a parent chose not to allow her/his child to participate in the trial, we removed her/his child's name from the testing rolls.

We inform children during testing in both trial arms that all tests are voluntary and that they may opt out of the test if they choose to. We do not seek written consent from parents or children. The "opt-out" method of parental permission is considered to be an ethical way of informing participants in low-risk interventions. Such procedures, when compared to seeking active consent, reduce time needed to seek consent and may avoid significant sampling bias and under-reporting [18].

There is a risk of sample selection bias if participation/consent decisions are different for parents, teachers, or Panchayat leaders in controls versus intervention clusters for the STRIPES trial. As mentioned previously, we provide small incentives to schools and children that participate in order to encourage parents and teachers to participate and reduce biased post-randomisation sample attrition.

Analysis strategies

The main comparisons will be as follows.

Comparison 1: eligible children in eligible villages allocated to any education intervention versus those in eligible villages not allocated to receive an education intervention

Comparison 2: eligible girls in eligible villages allocated to receive the education instruction and learning materials intervention versus those in eligible villages allocated to receive the education instruction, learning materials and school uniform and other 'kit'.

Programme cost per 0.1 standard deviation improvement in test scores will be calculated for both intervention groups.

Sample Size

On average 19 eligible children per eligible village took the baseline test. A recent study with a similar intervention in urban areas found that the average test score of children receiving additional instruction rose by 0.14 standard deviations compared to controls over a two year period [12]. Assuming that at least 15 children per village (80% of the number that turned up at the baseline test) will take the test at the end of the trial and an intra-cluster correlation coefficient of 0.03, then 107 intervention villages and 107 control villages will give over 90% power to detect a difference of 0.14 SD in the standardised score between intervention and control villages with a conventional 2-sided significance level of 5%.

Statistical Analysis

For both the primary and secondary comparisons mean child-specific composite test scores at the end of the second academic year will be compared using un-paired t-tests with robust standard errors to allow for

clustering by village. For the primary comparison an intervention-gender interaction will be tested.

The primary analysis will follow the intention to treat principle (i.e. the participants will remain in the group they were randomised to and not analysed according to the interventions actually received).

Further analysis will adjust for caste, education and other characteristics of parents, schools and villages. We will explore the possibility of carrying out a stratified analysis based on enumerated children with and without baseline test scores (adjusting for baseline in the strata with a baseline score). Analysis of covariance (with robust standard errors) will be used where baseline adjustment is required. Bootstrap confidence intervals will be reported for non-normally distributed continuous outcomes.

Administrative structures

Implementation team

The team responsible for implementing the education intervention will consist of:

- A Programme Coordinator, who will head the team and be responsible for the overall delivery of the intervention
- 13 field coordinators who will conduct on-the-ground supervision and will be responsible for monitoring the intervention
- Approximately 100 community activists who will be responsible for administering the supplementary teaching described above
- Trainers to train the coordinators and community activists

Research Team

The team responsible for collecting the data that will evaluate the effectiveness of the project will consist of:

- A project coordinator who will lead the team
- 5 team leaders who will supervise the test administration team
- 9 test administration teams, consisting of 3 test administrators each, to administer the baseline and endline tests

The research team will be formed and supervised by an external group that focuses on research implementation. The tests are designed by an external agency specializing in evaluating educational outcomes in India's primary schools.

The Trial will be jointly managed by the CHAMPION Trial Coordinator, the STRIPES Programme Coordinator and the CHAMPION Research Coordinator.

Steering committee

The steering committee will be formed by the Trial Coordinator and representatives from the Naandi Foundation, Effective Intervention and the London School of Hygiene and Tropical Medicine.

Data and safety monitoring committee

Since there is no risk to health or safety from the education initiatives in this trial, there will be no separate data monitoring committee (DMC). As this trial is conducted in conjunction with the CHAMPION trial and that has an independent DMC the data obtained from the STRIPES trial will be also presented to that committee at the end of the second test.

Data collection

In all eligible villages we will conduct two tests, a 'baseline' which was completed in the summer and autumn of 2008, before the intervention began, and an 'endline' that will be conducted in the spring of 2010, once the intervention has concluded. These tests have been designed by an Indian firm that specializes in designing assessments for understanding primary education levels in rural India. Baseline and endline tests were designed for children in each of the three standards, second, third and fourth, at the baseline by this firm. Each test is composed of two sections, mathematics and language. Each section has three types of question: one type of question which tests the specific competencies defined by the Andhra Pradesh State curriculum for that standard, a second type which tests competencies as defined by the Indian National curriculum for that standard, and a third type testing competencies that allow for comparison of test results with other evaluations conducted internationally. The composite score of the endline test serves as the primary endpoint for the study.

These tests are administered to all eligible children available in the village on the day of testing. In some cases, if a holiday, strike, or administrative necessity negatively impacts attendance at the test, a second visit to that village to reach all eligible and available children is conducted.

To collect background information on the family, a survey team will administer a survey to the parents or guardians of eligible children. This survey will include questions on socioeconomic status, education and literacy of parents, distance from home to fetch water and other factors with possible influence on the education level of the child. These data will be used in the secondary analyses to understand whether the intervention had a differential effect on various sub-groups identified by characteristics such as caste and income level.

Background information on each school and village will also be collected by the survey team. These data will include the number of girl and boy students in standards 2, 3 and 4 at each school in eligible villages, the number of teachers in each school, indicators of the quality of infrastructure at the school, such as number of blackboards and presence of toilets, and the caste

composition of the village. These data will be used for secondary analyses.

Ethical Approval

The CHAMPION protocol, which stated that the control group will have educational interventions using the Naandi Foundation's educational programme, has received ethical approval from LV Prasad Eye Institute, Hyderabad, India which is affiliated with the Indian Council of Medical Research (Reference number: LEC07002) and from the ethics committee of the London School of Hygiene and Tropical Medicine (Reference number: 5166).

Discussion

Economic evaluation and sustainability of measures after intervention

Along with the overall impact of the intervention, we will conduct an analysis of the costs of both interventions to compare the relative benefit of the base programme of tutoring and materials to that of the programme with the addition of kits for girls. We will also conduct a cost-effectiveness analysis in which we calculate the programme cost for 0.1 standard deviation improvement for both intervention arms.

Ethics/protection of human subjects

Other than the consent processes discussed above, there are no major ethical issues for this study over and above those already discussed for the parallel CHAMPION Trial.

Abbreviations

AP: Andhra Pradesh; ASER: Annual Status of Education Report; CA: Community Activist; DMC: Data Monitoring Committee; NGO: Non-Governmental Organization; RCT: Randomised Controlled Trial; SSA: Sarva Shiksha Abhiyan; TSC: Trial Steering Committee.

Acknowledgements

Chitra Jayanthi and Rohini Mukherjee provided invaluable counsel on the design of this study. Educational Initiatives designed the baseline test and GH consultants managed the data collection. Effective Intervention and the Naandi Foundation are the main sources of funding for this study.

Author details

[1]Effective Intervention, Centre for Economic Performance, London School of Economics, Houghton Street, London, WC2A 2AE, UK. [2]The London School of Hygiene and Tropical Medicine, Keppel Street, London WC1E 7HT, UK. [3]The Naandi Foundation, 502 Trendset Towers, Road Number 2, Banjara Hills, Hyderabad, 500034, India.

Authors' contributions

All authors contributed to the design of the study and have read, commented and approved the manuscript.

Competing interests

The Naandi Foundation is actively involved in administering education support programmes in several states across India. Those authors not affiliated with the Naandi Foundation have no competing interests to declare.

Received: 3 August 2009
Accepted: 1 February 2010 Published: 1 February 2010

References

1. Department of Higher Education, Ministry of Human Resource Development, Government of India: **Provisions of the constitution of India having a bearing on education.**http://www.education.nic.in/constitutional.asp#Fundamental, (Accessed April 24th, 2008).
2. Department of School Education and Literacy, Ministry of Human Resource Development, Government of India: *Sarva Shiksha Abiyan* http://ssa.nic.in/national-mission/sarva-shiksha-abhiyan-mission-statement, (Accessed December 8th, 2008).
3. Pratham. ASER: **Annual Status of Education Report.** *PRATHAM Resource Centre, New Delhi* 2007.
4. Registrar General and Census Commissioner: *Census of India, Provisional Population tables table 7, Paper 1 of 2001. Delhi* 2001http://www.censusindia.gov.in/Data_Products/Library/Provisional_Population_Total_link/webed.html, (Accessed December 8th, 2008).
5. UNESCO: *Education for All Global Monitoring Report* UNESCO Publishing. Paris 2006.
6. Reddy VR, Rao RN: **Primary education: progress and constraints.** *Economic and Political Weekly* 2003.
7. Kambhampti US, Pal S: **Role of Parental Literacy in Explaining Gender Difference: Evidence from Child Schooling in India.** *The European Journal of Development Research* 2001, **13**:97-119.
8. UNESCO: **Education For All Global Monitoring Report 2003/4, Gender and Education for All, The Leap to Equality.** UNESCO Publishing. Paris 2003.
9. Kingdon GG: **The Gender Gap in Educational Attainment in India: How much can be explained?.** *The Journal of Development Studies* 2002, **39**:25-53.
10. Dasgupta M: **Selective discrimination against female children in rural Punjab, India.** *Population and Development Review* 1987, **13**:77-100.
11. Herz B, Sperling GB: **What works in girls' education: evidence and policies from the developing world.** Council on Foreign Relations, Washington 2004.
12. Banerjee A, Cole S, Duflo E, Linden L: **Remedying Education: Evidence from Two Randomized Experiments in India.** *The Quarterly Journal of Economics* 2007, **122**:1235-1264.
13. Bjorkman M, Svensson J: **Power to the People: Evidence from a Randomized Field Experiment of a Community-Based Monitoring Project in Uganda.** Centre for Economic Policy Research Discussion Paper Series 2007; Paper No. 6344.
14. Jimenez E, Sawada Y: **Do Community-Managed Schools Work? An Evaluation of El Salvador's EDUCO Program.** *The World Bank Economic Review* 1999, **13**:415-441.
15. Glewwe P, Kremer M, Moulin S: *Textbooks and Test Scores: Evidence from a Prospective Evaluation in Kenya. Mimeo* Harvard University 2002.
16. Boone P, Mann V, Eble A, Mendiratta T, Mukherjee R, Figueiredo R, Jayanty C, Frost C, Padmanabh MR, Elbourne D: **Community health and medical provision: impact on neonates (the CHAMPION Trial).** *BMC Pediatrics* 2007, **7**:26.
17. Edwards S, Braunholtz D, Lilford R, Stevens A: **Ethical issues in the design and conduct of cluster randomised controlled trials.** *BMJ* 1999, **318**:1407-1409.
18. Moore L, Moore G, Tapper K, Lynch R, Desousa C, Hale J, Roberts C, Murphy S: **Free breakfasts in schools: design and conduct of a cluster randomised controlled trial of the Primary School Free Breakfast Initiative in Wales.** *BMC Public Health* 2007, **7**:258.

doi:10.1186/1745-6215-11-10
Cite this article as: Eble *et al.*: The STRIPES Trial - Support to Rural India's Public Education System. *Trials* 2010 **11**:10.
