# Supplementary material for: The Support to Rural India's Public Education System (STRIPES) Trial: A Cluster Randomised Controlled Trial of Supplementary Teaching, Learning Material and Material Support
Source: PLoS One. 2013 Jul 16;8(7):e65775. doi: 10.1371/journal.pone.0065775 (PMC3712986; doi:10.1371/journal.pone.0065775)
Supplement: Box S1 — Further details of intervention. (DOCX) [file pone.0065775.s004.docx]

**Box 1: Further details of intervention**

**Cooperative-Reflective Learning (CRL)**

To manage the multi-grade, multi-level remedial classes, Community Volunteers (CV) were trained in CRL pedagogy. CRL promotes learning through social interaction. Students of all levels of ability work together in structured groups toward a shared or common goal. It fosters peer learning, develops higher order thinking and leadership qualities, and makes children responsive to learning. The CRL pedagogy was used to reinforce the curriculum that children learnt in class during school hours and was tailored to students’ class-specific needs and learning levels. Teaching methods based on CRL included self-learning materials, use of a group leader to lead the group and group work.

**Supplementary Teaching & Learning Materials (TLM)**

CRL based TLM was used along with oral instruction at the remedial classes. TLM was designed to strengthen concept learning and problem solving. Children used these repeatedly to practice concepts to enhance clarity. TLM helped CVs explain language and mathematics concepts to the children and also helped in evaluating the improvement in children’s learning levels. CRL based TLM were interactive ensuring children’s participation, and gave them an opportunity to explore and arrive at solutions to the exercises. Consistent use of TLM in this way stimulated active learning in children ensuring, learning and retention. For STRIPES, TLM was prepared in Telugu, the regional language and the medium of instruction in the schools. The principal TLM were developed and tested by education experts from both the Naandi Foundation and external consultants, and were supplemented by the TLM developed by the intervention team at the field level. A bundle of learning materials, including a pen, four pencils, two notebooks, a ruler and an eraser, was provided to each participating child for use in these supplementary classes.
